# Long-term effects of mild traumatic brain injuries to oculomotor tracking performances and reaction times to simple environmental stimuli

**DOI:** 10.1038/s41598-018-22825-5

**Published:** 2018-03-15

**Authors:** Alessander Danna-Dos-Santos, Sambit Mohapatra, Maria Santos, Adriana M. Degani

**Affiliations:** 1 Dr. Charles T Leonard Motor Control Laboratory, University of Montana, Missoula, MT USA; 2 Neural Injury Center, University of Montana, Missoula, MT USA; 3 Department of Rehabilitation & Movement Sciences, University of Vermont, Burlington, VT USA

## Abstract

Understanding the long-term effects of concussive events remains a challenge for the development of modern medical practices and the prevention of recurrent traumas. In this study, we utilized indices of oculomotor performance and the ability to react to simple environmental stimuli to assess the long-term motor effects of traumatic brain injury in its mildest form (mTBI). We performed analysis of eye movement accuracy, investigated the presence of abnormal eye movements, and quantified time to react to simple environmental stimuli on long-term mTBI survivors. Results indicated the presence of impairments to basic neural functions used to explore and respond to environmental demands long after the occurrence of mTBIs. Specifically, the result revealed the presence of abnormal saccadic eye movements while performing horizontal smooth pursuit, diminished accuracy of primary saccadic horizontal eye movement, and a widespread slower reaction to both visual and auditory stimuli. The methodology used in this study indicated to be potentially useful in aiding future investigations of neural circuitry impaired by mTBI and provide indices of recovery in future clinical trials testing mTBI-related clinical interventions.

## Introduction

Mild traumatic brain injuries (mTBIs) are well recognized by their potential to lead to serious long-term neurological effects including impairments to cognitive functions, movement coordination, social behavior, and overall decrease in quality of life^1–4^. In fact, previous reports have linked the presence of higher rates of depression and dementia in populations with a history of concussion^5,6^. Considering its clinical and social significance, extensive efforts have been made to understand the neurophysiological mechanisms behind these symptoms as well as their progression over time. However, the complex interaction of all possible symptoms following single or multiple mTBIs has limited the development of efficient methods of assessment across all cases.

Extensive neuropsychological testing procedures have been used to assess the effects of mTBIs. Despite being valid, results produced by these methods may be influenced by motivational factors, practice effects, baseline intelligence, and cognitive fatigue produced by long testing sessions^7–9^. Interestingly, detection of brain lesions through imaging techniques has shown poor correlation with clinical symptoms and their severity^10,11^. Recently, it has been proposed that measures of basic neurophysiological functions of sensory-motor integration are a potential approach to uncovering the presence of fundamental neurological abnormalities caused by mTBI. This consideration is based on the rationale that planning, implementing, and adapting motor actions is dependent on a broad ensemble of neural mechanisms spanned across multiple areas of the CNS including cortical and subcortical pathways^12–14^. Therefore, even subtle impairments of these mechanisms caused by neural insults are likely to result in abnormal patterns of movement control, including eye tracking abilities and the ability to react to environmental stimuli. Performances of this nature are quantifiable, less influenced by patient’s subjectivity, and fit well as an aid to the current standard neuropsychological procedures.

The application of principles of sensory-motor integration in laboratory settings is not new, but recent technical developments have caused these techniques to become more sensitive and reliable^15–17^. For example, the use of these principles has allowed the detection of subtle balance impairments in long-term mTBI survivors^18^ and abnormalities in coordination of multi-finger force production and control in Parkinson’s disease^19,20^. Studies utilizing indices of oculomotor performance have been no exception; however, its application to cases of mTBI has yielded conflicting outcomes^8,21,22^. For example, Kraus *et al*.^22^ found that visually guided saccadic tasks showed longer latencies and reduced accuracy irrespective of the severity of TBI, whereas neuropsychological testing was only impaired in the moderate to severe individuals with TBI. Similarly, Suh *et al*.^23^ investigated smooth pursuits and reported decreased target prediction in mTBI primarily due to increased eye position error and variability, but they failed to see general oculomotor impairment (eye gain) or reduced IQ in mTBI.

One important factor to consider as a potential cause for this broad range of outcomes is the methodology used in these studies, including their inclusion/exclusion criteria, discrepancies in analytical procedures, and sensitivity of metrics of interest. Our study was designed to address the feasibility of oculomotor performance and one’s ability to react to simple environmental stimuli to distinguish the presence of long-term effects of traumatic brain injury in its mildest form. Here we assessed patterns of oculomotor tracking while performing horizontal smooth pursuit tasks, horizontal saccadic movements, and simple reaction times to visual and auditory stimuli. We hypothesized that long-term effects of mTBI will include significant reductions in eye-tracking accuracy and a widespread delay in reaction times to both auditory and visual stimuli.

## Results

### Performance of Horizontal Smooth Pursuit at 0.10 Hz

**Figure 1:**
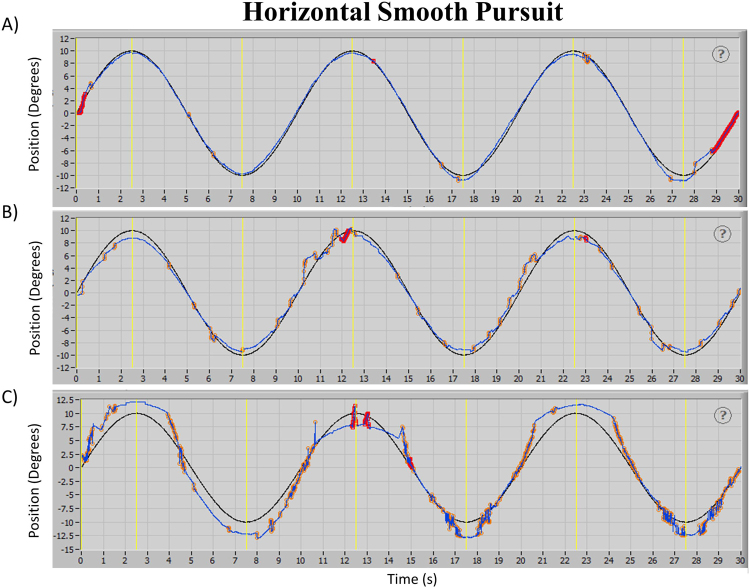
Actual display of recorded eye angular displacement in the horizontal axis from a participant of Control group (panel A) and two participants of mTBI group (panels B and C) performing the horizontal smooth pursuit task. Left and right eye displacements are combined and shown by the blue trace. Black trace shows angular position of stimulus presented. Orange markers identify the presence of saccadic ocular movements while red markers identify data excluded from analysis.

Figure 1 shows actual recordings of the combined eye angular displacement (blue trace) of three participants during the execution of this task. Recordings obtained from a control participant are displayed in panel A, while panels B and C show the recordings from two participants with a history of mTBI. Note that oculomotor performance on panel B is easily discernible from the control by its larger deviation of the eyes from the target (black trace). This inaccuracy was accompanied by abnormal high-speed short shifts (orange markers) recognized as intrusive abnormal saccadic movements. This later effect was also present in recordings shown in panel C. The presence of these two characteristics was found to be a significant discriminating factor between the two groups studied (Controls *vs* mTBI). Specifically, mTBI participants were found to be significantly less accurate in following the target (*p* = 0.0289) and presented with a significantly larger presence of intrusive saccades (*p* < 0.0001). No significant indications of phase shift between the target and the eyes or significant asymmetries were found between the two groups (*p* = 0.5064 and *p* = 0.4046, respectively). Table 1 shows medians and quartiles recorded for each variable of interest and the two groups.

**Table 1:** Medians and quartiles (25^th^ and 75^th^) across participants from Control and mTBI groups for variables of interest recorded during the execution of the horizontal smooth pursuit task at 0.10 Hz.

	*Control* Median (25^th^; 75^th^ Quart.)	*mTBI* Median (25^th^; 75^th^ Quart.)	Mann–Whitney U tests (*p*-value)
*Accuracy Smooth Pursuit* ^a^	0.03 (0.01; 0.05)	0.06 (0.02; 0.09)	0.0600
*Asymmetry* (%)	0.26 (−0.54; 0.99)	0.36 (−0.58; 2.13)	0.5064
*Phase* (°)	0.32 (−0.13; 0.78)	0.35 (−0.18; 1.32)	0.4046
*Intrusive Saccades* (%)	17.56 (11.62; 24.34)	32.98 (23.51; 51.20)	**0.0000**

^a^*Accuracy Smooth Pursuit* was defined as the absolute error obtained between the averaged gain values computed between the slow phase component of eye velocity and the pursuit tracker stimuli. Perfect accuracy is evidenced when *Accuracy Smooth Pursuit* is equal to zero, while any positive value (*Accuracy Smooth Pursuit* > 0) indicates less accurate tracking.

### Performance of Horizontal Saccadic Movements

**Figure 2:**
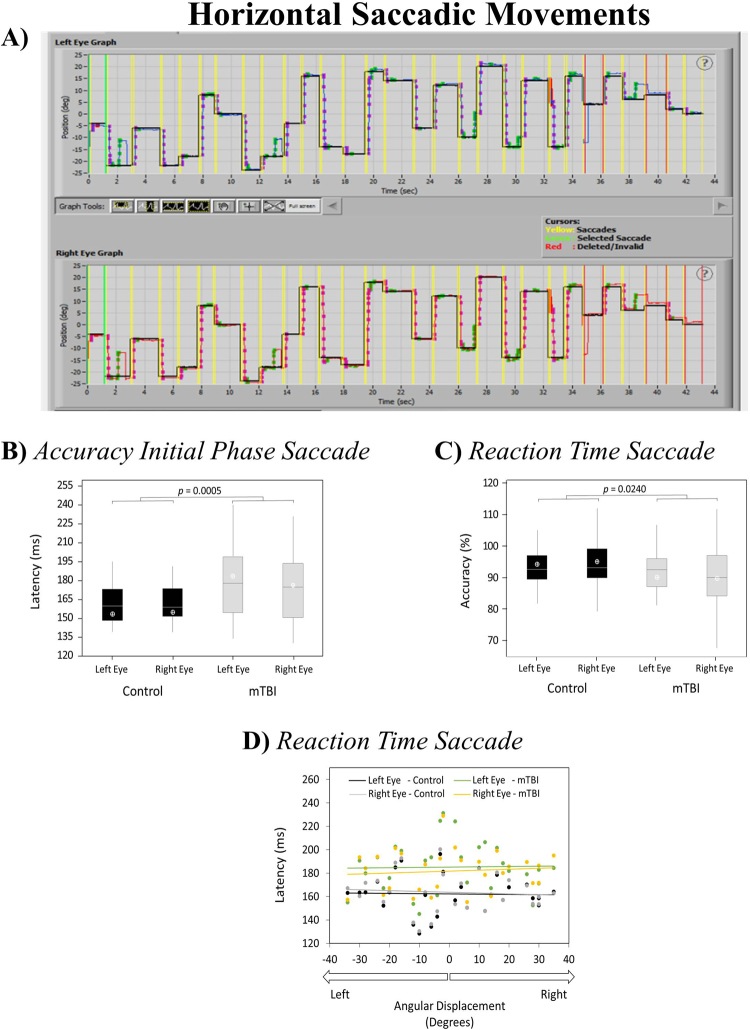
Actual display of recorded left and right eye angular displacement in the horizontal axis from a participant from the Control group (black trace shoes angular position of stimulus presented) (panel A); boxplots from *Accuracy Initial Phase Saccade* and *Reaction Time Saccade* recordings obtained for each eye across participants (panels B and C, respectively); and averages across participants for *Reaction Time Saccade* obtained for all angular displacements (panel D) recorded during the horizontal saccadic movement task. Note: ^1^Averages across participants in panels B and C are indicated by the white open circles. ^2^Traces in panel D represent the linear regression calculated for *Angular displacements vs Reaction Time Saccade*.

Figure 2 (panel A) shows an actual recording of a control participant performing the horizontal saccadic task. Overall accuracy of saccadic movements was found to be similar between the two experimental groups and no asymmetries between right and left eyes were found within any of the groups (p > 0.05). However, a significant reduction of accuracy was found for the initial phase of the saccadic movement (Fig. 2, panel B). Additionally, a significant increase in the time to initiate the saccadic movement (*Reaction Time Saccade*) was recorded for the mTBI group (Fig. 2, panel C) and confirmed by a series of four independent linear regressions fitted with *Reaction Time Saccade* and across angular displacements (Fig. 2, panel D) (R^2^ values = 0.0008 [Control group – left eye, black trace], 0.0074 [Control group – right eye, grey trace], 0.0006 [mTBI – left eye, green trace], and 0.0089 [mTBI – right eye, orange trace]). No significant differences were found between right and left eyes between the mTBI and healthy controls (Fig. 2, panels A and B). Table 2 shows medians and quartiles recorded for each variable of interest across participants.

**Table 2:** Medians and quartiles (25^th^ and 75^th^) across participants from Control and mTBI groups for variables of interest recorded during the execution of the horizontal saccadic movements task.

	*Control* Median (25^th^; 75^th^ Quart.)	*mTBI* Median (25^th^; 75^th^ Quart.)	Mann–Whitney U tests (*p*-value)
*Reaction Time Saccade* – left eye (ms)	160.0 (148.4; 173.2)	177.9 (154.7; 198.6)	**0.0042**
*Reaction Time Saccade* – right eye (ms)	159.1 (151.6; 173.8)	174.9 (150.5; 193.6)	**0.0481**
*Reaction time Saccade* – combined (ms)	159.4 (150.2; 173.8)	176.0 (151.3; 196.2)	**0.0005**
*Accuracy Initial Phase Saccade* – left eye (%)	92.6 (89.4; 97.0)	92.5 (87.1; 96.0)	0.5067
*Accuracy Initial Phase Saccade* – right eye (%)	93.2 (89.8; 99.1)	90.0 (84.2; 97.1)	0.1260
*Accuracy Initial Phase Saccade* – combined (%)	92.7 (89.7; 97.6)	91.7 (85.6; 96.2)	**0.0240**
*Overall Accuracy Saccade* – left eye (%)	97.26 (94.3; 101.7)	99.4 (95.2; 106.1)	0.2195
*Overall Accuracy Saccade* – right eye (%)	95.0 (92.9; 103.0)	98.2 (90.15 ;103.5)	0.8807
*Overall Accuracy Saccade* – combined (%)	96.8 (93.5; 101.6)	98.5 (93.0; 104.6)	0.2651

### Reaction time to single environmental stimuli (visual and auditory)

**Figure 3:**
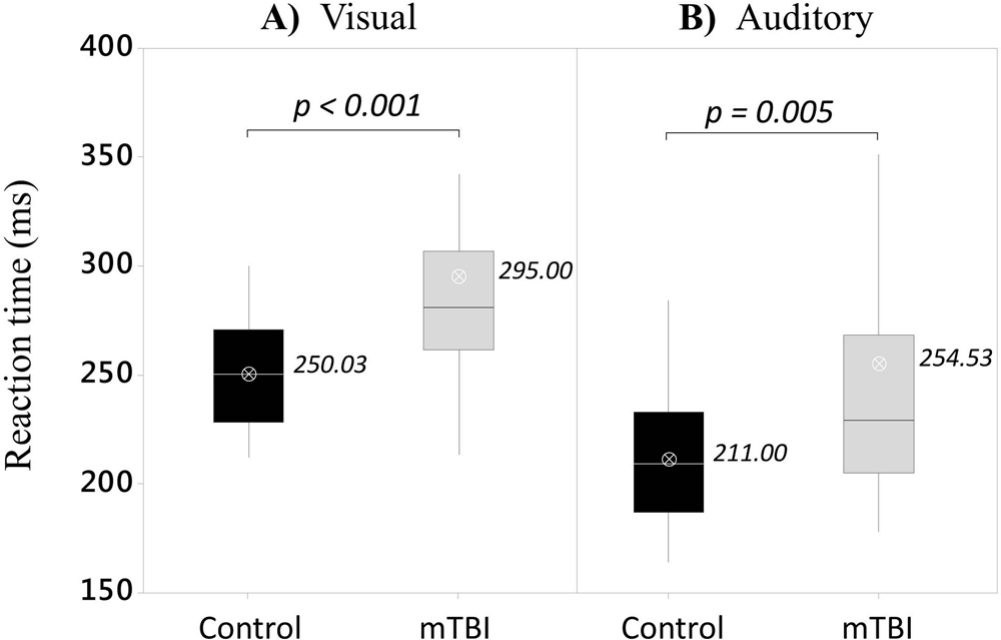
Boxplots from reaction times recorded for simple visual and auditory conditions (panels A and B, respectively) for both experimental groups (Control and mTBI). Averages across participants are indicated by the white open circles.

Figure 3 shows the medians, quartiles, and averages recorded for the two experimental groups and conditions (Visual and Auditory). Average delays of 44.97 ms and 43.53 ms were found in the mTBI group when compared to healthy controls for both visual (*M* = *250.03 ms, SD* = *24.85 ms)* and auditory reaction times (*M* = *211.00 ms, SD* = *31.18 ms)*. These delays were confirmed to be significant by two independent Mann-Whitney U tests ran on *Reaction Time Visual* and *Reaction Time Auditory* indices (*p* < 0.001 and *p* = 0.0055, respectively). Table 3 show medians and quartiles recorded for *Reaction Time Visual* and *Reaction Time Auditory* across participants.

**Table 3:** Medians and quartiles (25^th^ and 75^th^) across participants from Control and mTBI groups for variables of interest recorded during the execution of the visual and auditory reaction time tasks.

	*Control* Median (25^th^; 75^th^ Quart.)	*mTBI* Median (25^th^; 75^th^ Quart.)	Mann-Whitney U tests (*p*-value)
*Reaction Time Visual* (ms)	250 (228; 270)	281 (262; 306)	**0.0000**
*Reaction Time Auditory* (ms)	209 (187; 231)	229 (206; 264)	**0.0055**

### Neurobehavioral Symptoms Inventory

Recording of current symptoms allowed us to confirm the presence of long-term effects following mTBIs. Table 4 shows averages and standard deviation of scores obtained for the two groups from self-reports of symptoms felt along two weeks prior to the experimental session. Multiple symptoms affected our mTBI cohort including difficulties falling or staying asleep, headaches, irritability, poor concentration, difficulty remembering information and making decisions, slowed thinking, poor coordination, changes in appetite, and hearing and vision difficulties. These findings were confirmed by a series of two-tailed student’s T tests (Table 4). A significant increase in the total scores of neurobehavioral symptoms recorded for the mTBI participants was found (*p* = 0.0015).

**Table 4:** Averages and standard deviations across participants from Control and mTBI groups of scores recorded by the Neurobehavioral Symptoms Inventory.

	*Control* Average ± SD	*mTBI* Average ± SD	*T*-test (*p*-value)
Difficulty falling or staying sleep	0.44 ± 0.65	1.24 ± 1.21	**0.0028**
Headaches	0.39 ± 0.60	1.14 ± 0.99	**0.0009**
Irritability	0.31 ± 0.52	1.03 ± 1.21	**0.0045**
Feeling anxious or tense	0.53 ± 0.70	1.00 ± 1.16	0.0610
Poor concentration	0.19 ± 0.47	1.24 ± 1.21	**0.0001**
Fatigue, loss of energy	0.44 ± 0.73	0.93 ± 1.22	0.0659
Cannot remember things	0.25 ± 0.50	1.03 ± 1.05	**0.0007**
Slowed thinking, difficulty getting organized	0.14 ± 0.42	0.90 ± 1.01	**0.0006**
Poor frustration tolerance	0.47 ± 0.81	0.76 ± 1.12	0.2545
Feeling depressed or sad	0.23 ± 0.43	0.69 ± 1.17	0.0516
Difficulty making decisions	0.11 ± 0.32	0.86 ± 1.06	**0.0008**
Hearing difficulty	0.08 ± 0.28	0.66 ± 1.01	**0.0058**
Vision problems	0.19 ± 0.47	0.55 ± 0.91	0.0620
Sensitivity to light	0.22 ± 0.83	0.55 ± 0.83	0.1166
Poor coordination	0.11 ± 0.32	0.55 ± 0.83	**0.0104**
Loss of appetite/Increase in appetite	0.14 ± 0.68	0.46 ± 0.79	0.0894
Loss of balance	0.14 ± 0.35	0.38 ± 0.86	0.1672
Feeling dizzy	0.08 ± 0.37	0.31 ± 0.66	0.1053
Sensitivity to noise	0.00 ± 0.00	0.41 ± 1.05	0.0433
Numbness or tingling	0.17 ± 0.45	0.31 ± 0.89	0.4331
Nausea	0.19 ± 0.71	0.14 ± 0.44	0.6959
Change in taste and/or smell	0.03 ± 0.17	0.21 ± 0.68	0.1730
Total Score	4.86 ± 5.98	15.34 ± 15.53	**0.0016**

The Neurobehavioral Symptoms Inventory includes 22 questions. All units reported in this table are arbitrary units (from 0 to 4) representing the severity of the symptoms listed within the last two weeks pre-test where: 0 = None (rarely if ever present); 1 = Mild (occasionally present and does not disrupt daily activities); 2 = Moderate (often present and occasionally disrupts daily activities); 3 = Severe (frequently present and disrupts daily activities); 4 = Very Severe (almost always present resulting in inability to perform at work, school, or home).

## Discussion

This study was designed to employ indices of oculomotor performance and one’s ability to react to simple environmental stimuli to identify the presence of impairments of these functions long after a mild traumatic brain injury (mTBI). Overall, our hypotheses were supported and our results provide evidence of specific changes in the measures of eye tracking accuracy, widespread delays in reaction times, and significant adaptations to normal patterns of eye tracking movements. These findings are in line with actual neurobehavioral symptoms reported by the mTBI group.

The ability to track objects in the environment is an important feature for humans to interact with their surroundings. In particular, the ability to recognize the presence of an environmental hazard is directly linked to our ability to fix our gaze on a target, recognize the threat, and implement a plan of action. Therefore, the central nervous system (CNS) is imposed with a series of tasks and time constraints that require a harmonic integration of several neural centers located in multiple regions and linked through an efficient transmission of information. Results uncovered in the present study suggest CNS impairments in individuals with mTBIs long after the last traumatic episode. Although this suggestion has been offered by previous studies, the metrics presented here are relatively easier to obtain for clinical use that represents a significant step forward in the diagnosis and monitoring of recovery rates.

Saccadic intrusions (irregular episodic occurrences of fast eye movements) are classified according to whether or not the intrusive saccades are separated by a brief interval in which the eyes are stationary. Until recently, significant alterations in reflexive saccades have only been demonstrated in severe TBI^9^. Complex saccade measurements, such as anti-saccades, have shown saccade alteration in mTBI^24^. Pearson and colleagues^25^ found saccadic reaction times were delayed in mild TBI in a completely reversible manner following boxing. Mullen *et al*.^26^ confirmed that saccadic reaction times are prolonged in mTBI patients within one week following injury but resuming to normal levels three weeks after injury. Heitger *et al*.^27^ reported no significant difference in saccadic reaction time measured approximately four to five months following injury between cases of mTBI with prolonged symptoms and patients who had recovered.

In the present study, the mTBI group revealed a significant larger presence of saccadic intrusions during the execution of smooth pursuit several months after the last injury. Even though the presence of these saccadic intrusions (<20%) are considered to be beneficial to correct velocity mismatches between eye and target, its excessive presence can be detrimental for maintenance of gaze fixation and image interpretation^28^. For example, the excessive presence of saccadic movements during smooth pursuit can potentially shorten fixation times and interfere with semantic recognition and interpretation. This is due to erratic eye movements that drives gaze away from the intended targets too often. Similarly, the excessive presence of these saccadic intrusions can interfere with someone’s ability to judge the speed of approximation of an object, increasing the chance of new traumatic events. Although this study was not designed to test such hypotheses, our results show the potential for this type of testing to be used clinically to aid the diagnostics of late impairments of concussive syndrome.

It is important to emphasize that our mTBI cohort reported the presence of visual problems (often referred to as visual discomfort) in the Neurobehavioral Symptoms Inventory. Despite the absence of an in-depth optometric evaluation in our methodology, all volunteers wore their corrective lenses during experimental sessions. In addition, any volunteers showing signs of impaired visual acuity during the experimental sessions (e.g., double or smeared vision of the target, or impairment of depth perception) were not included in this study. Taken together, these observations allow us to suggest that the visual discomfort reported may arise from impaired abilities in gaze fixation rather than from inability to focus an image on the retinal surface.

In this study, we also found that mTBI survivors took a longer time (longer latencies) when reacting to simple environmental stimuli. In fact, all three indices recorded (*Reaction Time Visual*, *Reaction Time Auditory*, and *Reaction Time Saccade*) were found to be significantly longer in individuals with a history of mTBI when compared to the healthy controls. These observations suggest a widespread impairment in either processing or conduction of information across systems involved in the process of sensory-motor integration. For example, considering the lack of anatomical abnormalities in many of mTBI cases^11,29^, motor impairments resulting from mTBI may result from deficits rooted to the system of conduction, by which neural centers exchange information during the process of sensory-motor integration. This point of view has gained support from contemporary observations^30,31^.

Previous studies have shown that decrease in reaction time is closely linked with anatomical abnormalities in the corpus callosum (CC) and the superior longitudinal fasciculus (SLF). For example, Hulkover *et al*.^31^ compiled results from a large cohort of 100 studies that utilized MRI images (e.g., fractional anisotropy). The results suggested the existence of white-matter abnormalities in important areas for the integration of neural information within and between the two brain hemispheres. In fact, this study revealed the CC and both the superior and inferior longitudinal fascicules as the most common locations for abnormal readings of fractional anisotropy. This finding is of interest because the CC has recently been linked to its role in successful execution of bimanual coordination during motor actions^32–34^. According to Shen *et al*.^34^, a stable interaction of the two hemispheres is achieved thorough the homotopic projections of the CC, and movement coordination is then optimized. Caeyenberghs *et al*.^32^ studied survivors of closed head traumas, and these survivors revealed significant lower values of fractional anisotropy in the CC fibers connecting both pre-frontal cortices, both primary sensory cortices, and parietal cortical areas. They also exhibited significant slower movement time, longer time to switch to a new motor task, and poorer performance during the execution of the Purdue Pegboard test.

In addition to the CC, the SLF is also considered an important intra-hemisphere association pathway establishing connections between postrolandic regions and the frontal lobe^35,36^. This pathway is usually differentiated into four bundles of white matter conveying important information about the perception of the visual space (SFL subdivision II, SFL-II). This type of perception has been considered of importance to the organization of motor commands in humans due to its ability to provide a reliable neural representation of body configuration with regard to the external environment^37–41^. Recently, Danna-dos-Santos *et al*.^17^ showed that even a short-term absence of visual information could generate changes in neural mechanisms possibly related to the formation of multi-muscle synergies among postural muscles in healthy adults. Additionally, injuries to the caudal parietal area (SFL-II posterior origin) have been recognized as the possible cause for severe impairment in spatial attention^42^, while long-term mTBI survivors (up to 12 months post single head trauma) have consistently exhibited significant delays to recognize simple visual patterns when compared to healthy controls^43^.

It is also important to note that delayed reaction time may also occur due to impaired abilities of certain brain centers to elaborate responses. Lew *et al*.^44^ reported that individuals with TBI demonstrated significantly poorer performance in electrophysiological responses including diminished amplitudes and prolonged latencies in *P*_300_ responses. This observation indicates impaired organization and categorization of incoming sensory information, and prolonged behavioral reaction times that suggests slowing in the response execution process. Madigan *et al*.^45^ demonstrated modality (visual and auditory) effects on speed of information processing performance in individuals with moderate to severe TBI. More specifically, their report provides evidence of a disproportionately slower rate on the auditory task, relative to the visual task, followed by TBI. This finding is particularly consistent with the results produced by our study where the auditory reaction time were found to have longer delays than visual reaction times. Our finding is unique as we found slower reactions to be present in mild cases.

There are several possible explanations exist regarding the disproportionate slowing effect seen in the mTBI group. First, it may be that the auditory system is more vulnerable to damage than the visual system following a traumatic event. Temporal cortical regions, along with frontal regions, are especially vulnerable to insult in head injury, due to the proximity of these brain regions with bony skull protrusions and the mechanical forces involved in decelerating injuries^46^. Given the role of temporal regions in auditory functions, this explanation may account for the greater slowing observed in the TBI group on the auditory task. A second possible explanation is that the auditory and visual systems are differentially related to two distinct working memory systems. Baddeley’s model of working memory suggests the existence of a central executive system controlling two systems processing modality-specific information in working memory—the phonological loop and the visuospatial sketchpad^47^. Thus, following a traumatic insult, there may be a greater deficiency in the auditory component of working memory and a relative sparing of the visuospatial component.

A recent study by Ettenhoffer and Bardy^48^ has also uncovered significant increased latencies to the initiation of saccadic movements on long-term mTBI participants. This effect was found to be extensively more prevalent when compared to control participants, in particular, to those whom mTBI were more susceptible to attentional interferences. Their results also suggested that delays of this nature may be suggestive of difficulties with sustained attention and temporal anticipation of visual targets, supporting the rationale that their interaction with environmental hazards may be impaired when compared to healthy controls. Ettenhoffer and Bardy^48^ also demonstrated that initial information oculomotor impairments appear to be more common among those with a history of multiple mTBIs and those reporting high levels of chronic symptoms. These latter observations are particularly important to our results since our sample represents an mTBI group with an average of two or more traumatic episodes, and therefore, a potential cumulative impact of concussive events may have improved our ability to capture the impairments found in our study.

Lastly, the present study showed that individuals with mTBI also revealed a significant reduction on the displacement of the initial phase of the saccadic movement without significant changes in the final accuracy of these movements. Together, these findings suggest a potential change in movement strategy to perform gaze shifts where accuracy becomes more dependent on the correctional (secondary) phase of the saccadic movement. Similarly, diminished accuracy in ballistic saccadic movements has been reported in previous studies. Cifu *et al*.^8^ studied components of volitional saccadic movements and found mTBI survivors shift their gaze at slower speeds and similar reduction of displacement on its primary phase. However, contrary to our findings, the same authors reported a reduced accuracy on the overall gaze shift. This contradiction may be related to differences in average time from the last traumatic event among studies. In this present study, participants were exposed to their last traumatic event much longer than participants from other studies, which may have allowed a potential recovery of final accuracy.

## Materials and Methods

### Participants

Seventy-two volunteers participated in this study (*M* = 25.7 years of age; *SD* = 8.0 years) and all provided their informed consent based on procedures approved by the Institutional Review Board of the University of Montana (IRB protocol #165-15) and conforming to the Declaration of Helsinki.

An experimental group *(mTBI)* was formed by thirty-six individuals (twenty-two females and fourteen males; *M* = 26.6 years of age; *SD* = 9.2 years) with a history of single or multiple mild traumatic brain events. This cohort included traumatic occurrences from sports related activities, motor vehicle accidents, and military operations. Thirty-three individuals suffered mTBIs from direct impact and eight from blast induced traumatic events^49^. The average number of previous concussive events was 2.47 (*SD* = 1.95), and the average time elapsed between the last traumatic episode was 43.11 (*SD* = 52.45) months. Inclusion criteria for this group followed the standard classification proposed by the American Psychiatric Association as published in the Diagnostic and Statistical Manual of Mental Disorders^50^ and included (a) mTBI caused by either direct impact or blast-induced closed head trauma; and (b) presence of one or more of the following manifestations after the mTBI episode: confusion or disorientation, loss of consciousness for 30 minutes or less, post-traumatic amnesia for less than 24 hours, Glasgow Coma Scale score of 13–15 after 30 minutes post-injury or later on presentation for health care, and/or other transient neurological abnormalities such as focal signs, seizure, and intracranial lesion not requiring surgery. Exclusion criteria included the following: (a) manifestations of mTBI caused by drugs, alcohol, medications, and other injuries or treatment for other injuries (e.g., systemic injuries, facial injuries, or intubation); (b) other potentially confounding issues (e.g., psychological trauma, language barriers, or coexisting medical conditions); (c) history of penetrating cranial injury; (d) history of any type of neurosurgery; and (e) abnormalities of cranial nerve functions.

A control group (*Control*) was formed by thirty-six healthy individuals (twenty-four females and twelve males; *M* = 24.8 years of age; *SD* = 6.6 years). The exclusion criteria for the control group included the following: (a) previous history of head trauma, traumatic brain injury (including cerebral vascular accident), epilepsy, or seizure disorders; (b) history of substance abuse (drugs, alcohol, or controlled medication); (c) symptoms of psychological trauma such as panic attacks, frequent nightmares, insomnia, loss of self-esteem, anxiety, anger, frequent depression, feelings of despair, or emotional detachment; (d) previous history of peripheral neuropathy (including loss/decreased sensory or motor function in the upper extremity), acute upper extremity injury, recurring and/or unexplained headaches, cardiac pacemaker, pulmonary disease, or metallic implants in head, spine, or upper extremity; (e) history of any type of neurosurgery; and (f) abnormalities of cranial nerve functions. The ratio between males and females between groups (*Control* and *mTBI*) were found to be comparable (*X*^2^ (1) = 0.113, *p* = 0.737). Mean ages were also found to be comparable (*t*(63) = 0.96, *p* = 0.342).

### Materials and Procedures

An I-Portal system and VEST Neuro-Otologic Analysis Software (Neurokinetics, USA) were utilized to record and analyse all variables of interest. Prior to data recording, each participant went through a general neurological screening, then provided self-reports utilizing Neurobehavioral Symptom Inventory (NSI) to record their prevailing current symptoms. No time constraints for providing NSI information were stipulated. Participants who met the established inclusion and exclusion criteria were tested in an area free of noise and distractions.

**Figure 4:**
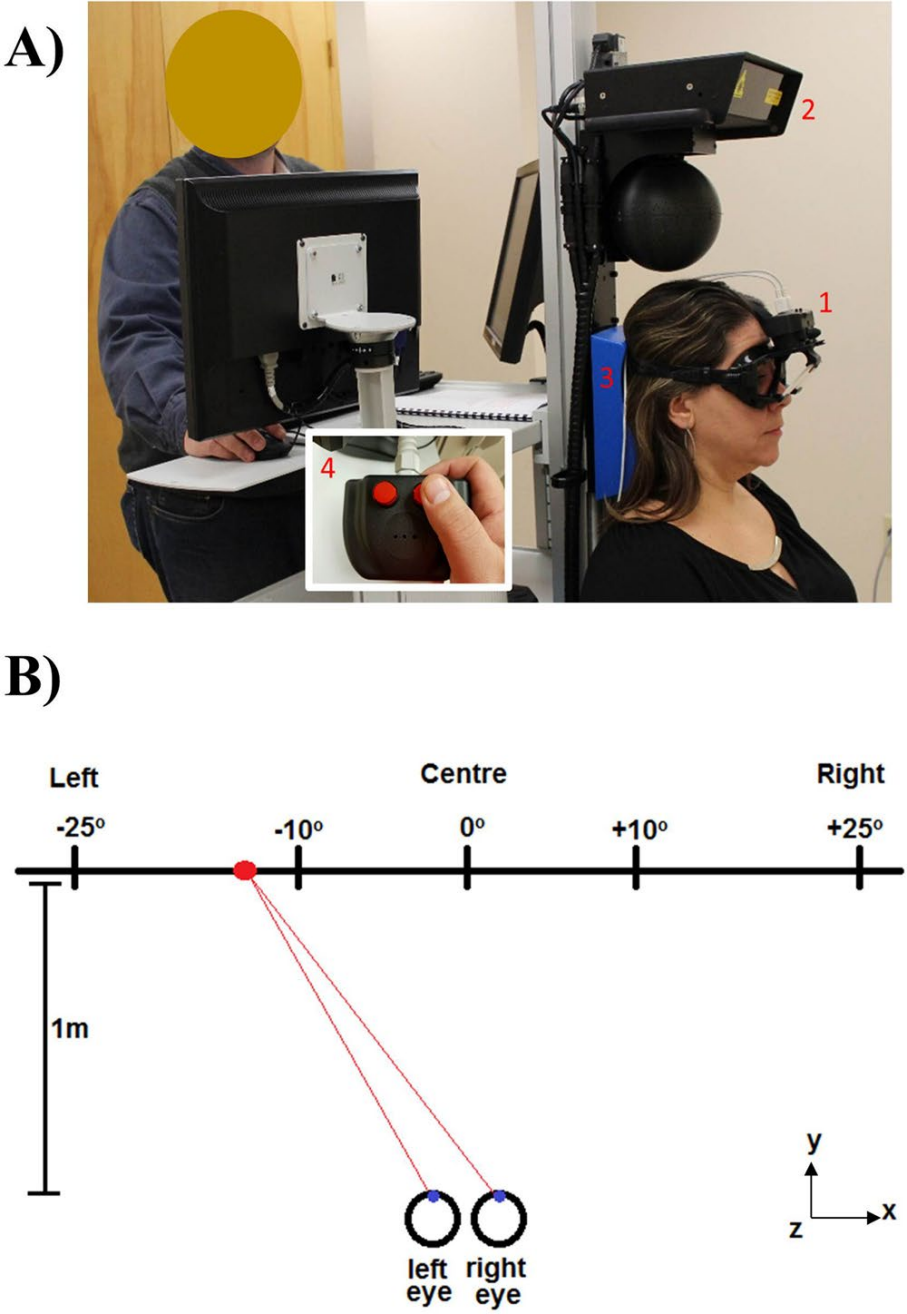
Example of positioning during data recording (panel A). (1) Eyewear with embedded cameras, (2) Laser emitter, (3) Head rest, and (4) Remote control used for the simple reaction time trials. Schematic representation of superior view of stimuli presentation and participant’s gaze (panel B). Sizes and proportions of figure components have been modified for illustration purposes.

Figure 4 (panel A) illustrates the experimental set-up. Participants remained seated for the whole experimental session while the positional coordinates of pupils of both eyes were recorded at a rate of 100 Hz by two dedicated cameras embedded in customized goggles. These coordinates were used to calculate the angular position of each eye with respect to the center of the visual field. Visual stimuli were applied by projecting a monochromatic laser (wavelength of 671 nm) target at eye level on a white plane surface 1 meter from the participants in a dark room. The laser emitter was securely placed to a vertical column localized behind the participant with a dedicated servomotor to allow presentation of the target on multiple areas of the visual field. The laser emitter and all cameras were calibrated to the same space of the visual field: a 90 cm × 90 cm (width x height) area with its center coinciding with the center of the visual field.

During the performance of both smooth pursuit and saccadic tasks, all participants were instructed to track the projected laser target with their eyes while head movements were not allowed. A moulded headrest was used to support, align, and constraint the participant’s head. During the execution of both visual and auditory simple reaction time tasks, a simple two-buttons remote control (Fig. 4, panel A) was used. Participants were instructed to use their dominant hand to press on one of the buttons as fast as possible after the recognition of the stimulus. During the entire recording session, the lights of the testing room remained off to allow optimal target presentation and no ambience distraction.

### Protocol and Variables of Interest

The experimental protocol was initiated by the calibration of recording systems and the laser emitter to the center of the visual field. The following experimental procedures were then conducted:

1. *Performance of Horizontal Smooth Pursuit*. A continuous visual stimulus (a projected laser target) was presented along the horizontal line of sight within a range of 20° (−10° to 10°; Fig. 4, panel B). Participants were instructed to focus their gaze at the stimulus at all times and track its displacement along the entire range as close as possible until the completion of twelve full cycles. The frequency of each cycle was 0.10 Hz. Variables of interest included the overall accuracy of smooth pursuit on keeping eyes on the target *(Accuracy Smooth Pursuit)* and the presence of intrusive saccadic eye movements *(Intrusive Saccades). Accuracy Smooth Pursuit* was defined as the absolute error obtained between the averaged gain values computed between the slow phase component of eye velocity and the pursuit tracker stimuli. Perfect accuracy is evidenced when *Accuracy Smooth Pursuit* is equal to zero, while any positive value (*Accuracy Smooth Pursuit* > 0) indicates less accurate tracking*. Intrusive Saccades* was defined as the relationship between the resulting summed times recorded from all saccadic intrusions with respect to the total tracking time. *Intrusive Saccades* was expressed in percentage (%).
2. *Performance of Horizontal Saccadic Movements*. A series of twenty-nine single visual stimuli was presented independently along the horizontal line of sight within a range of 45° (Fig. 4, panel B). Gaze angular displacement randomly varied from 2° to 35° in both right and left directions. Each stimulus consisted of a single laser point projection lasting 3 s when participants were asked to focus their gaze directly at the target until another stimulus was presented. When presented with a subsequent target, participants shifted their gaze as fast and as accurately as possible. Variables of interest included the time elapsed from stimulus presentation to the initiation of gaze shifting (*Reaction Time Saccade*), the accuracy of initial phase of horizontal saccade *(Accuracy Initial Phase Saccade)*, and the overall accuracy of saccadic movement to reach the target (*Overall Accuracy Saccade)*.
3. *Reaction time to single visual stimuli*. A series of ten single visual stimuli was presented independently and at the center of the visual field. Each stimulus consisted of a single laser projection lasting 2 s. Participants were asked to react as fast as possible by pressing on an instrumented button (Fig. 4, panel A) with the thumb of their dominant hand. Time between stimuli randomly varied between 0.5 s and 3 s to avoid any anticipation. Index of interest represented the time elapsed from stimulus presentation to the time the button was pressed (*Reaction Time Visual*).
4. *Reaction time to single auditory stimuli*. A series of ten single-tone auditory stimuli was generated independently behind the participant and at a symmetrical distance from both ears. Each stimulus consisted of a loud computer beep lasting 0.2 s. Similar to the previous task, participants were asked to react as fast as possible by pressing on the same instrumented button (Fig. 4, panel A) with their thumb. The time between stimuli randomly varied between 0.5 s and 3 s to avoid any anticipatory effect. Variable of interest represented the time elapsed from stimulus presentation to the time the button was pressed (*Reaction Time Auditory*).

### Statistical analyses

A series of Kolmogorov-Smirnov tests was utilized to check the normality in the distribution of each set of variables recorded. Due to the lack of normality features in the distribution of multiple data sets, non-parametric tests were employed (Mann-Whitney U tests) to test potential statistical differences between the variables of interest (*Accuracy Smooth Pursuit*, *Intrusive Saccades*, *Reaction Time Saccade*, *Accuracy Initial Phase Saccade*, *Overall Accuracy Saccade*, *Reaction Time Visual*, and *Reaction Time Auditory*) recorded for both experimental groups. Two-tailed (non-paired) student’s t tests were performed to test for potential statistical differences on the severity of symptoms recorded by the Neurobehavioral Symptom Inventory between the two experimental groups. All statistical tests were performed using the Minitab statistics software version 17 (Minitab, USA) while keeping the level of significance at 5% (0.05).

## References

[1] Emanuelson I, Andersson HE, Bjorklund R, Stalhammar D (2003). Quality of life and post-concussion symptoms in adults after mild traumatic brain injury: A population-based study in western Sweden. Acta Neurologica Scandinavica.

[2] Iverson GL (2005). Outcome from mildtraumatic brain injury. Curr Opin Pychiatry.

[3] Carroll LJ (2004). Prognosis for mild traumatic brain injury: results of the WHO collaborating centre task force on mild traumatic brain injury. J Rehab Med.

[4] Scholten JD, Sayer NA, Vanderploeg RD, Bidelspach DE, Cifu DX (2012). Analysis of US Veterans Health Administration comprehensive evaluations for traumatic brain injury in operation enduring freedom and operation Iraqi freedom veterans. Brain Inj.

[5] Gavett BE (2010). Mild traumatic brain injury: a risk factor for neurodegeneration. Alzheim Res Ther.

[6] Plassman, B. L. *et al* Documented head injury in early adulthood and risk of Alzheimer’s disease and other dementias. *Neurology* Oct 24, 55(8),1158–66 (2000). 10.1212/wnl.55.8.1158 11071494

[7] Taylor BC (2012). Prevalence and costs of co-occurring traumatic brain injury with and without psychiatric disturbance and pain among Afghanistan and Iraq war Veteran VA users. Med Care.

[8] Cifu DX (2015). Differential Eye Movements in Mild Traumatic Brain Injury Versus Normal Controls. J of Head Trauma Rehab.

[9] Williams IM (1997). Cerebral control of saccades and neuropsychological test results after head injury. Journal of Clinical Neuroscience: Official Journal of the Neurosurgical Society of Australasia.

[10] Johansson, B., & Ronnback, L. Evaluation of the mental fatigue scale and its relation to cognitive and emotional functioning after traumatic brain injury or stroke. *Int J Phys Med Rehabil* **2**(182), 10.4172/2329-9096.1000182 (2014).

[11] Harad FT, Kerstein MD (1992). Inadequacy of bedside clinical indicators in identifying significant intracranial injury in trauma patients. J Trauma.

[12] Kashluba S, Hanks RA, Casey JE, Millis SR (2008). Neuropsychologic and functional outcome after complicated mild traumatic brain injury. Arch. Phys Med Rehab.

[13] Latash, M. L. *Synergy*. 51–162 (Oxford University Press, 2008).

[14] Kandel, E. R., Schwartz, J. H. & Jessel, T. M. *Principles of Neuroscience*. 653–673 (McGraw-Hill, 2000).

[15] Danna-dos-Santos A, Slomka K, Latash ML, Zatsiorsky VM (2007). Muscle modes and synergies during voluntary body sway. Exp Brain Res.

[16] Danna-dos-Santos A, Degani AM, Latash ML (2008). Flexible muscle modes and synergies in challenging whole-body tasks. Exp Brain Res.

[17] Danna-dos-Santos A (2015). The influence of visual information on multi-muscle control during quiet stance: A spectral analysis approach. Exp Brain Res.

[18] Degani AM (2017). The effects of mild traumatic brain injury on postural control. Brain Injury.

[19] Park, J., Jo, H. J., Lewis, M. M., Huang, X. & Latash, M. L. *Exp Brain Res* **231**, 51 10.1007/s00221-013-3665-3 (2013). 10.1007/s00221-013-3665-3 PMC3797845 23942616

[20] Lewis MM (2016). Synergy as a new and sensitive marker of basal ganglia dysfunction: A study of asymptomatic welders. NeuroToxicology.

[21] Sussman E, Ho A, Pendharkar A, Ghajar J (2016). Clinical evaluation of concussion: the evolving role of oculomotor assessments. Neurosurgical Focus.

[22] Kraus MF (2007). Oculomotor function in chronic traumatic brain injury. Cognitive & Behavioral Neurology.

[23] Suh M, Kolster R, Sarkar R, McCandliss B, Ghajar J (2006). Deficits in predictive smooth pursuit after mild traumatic brain injury. Neuroscience Letters.

[24] Heitger MH (2006). Motor deficits and recovery during the first year following mild closed head injury. Brain Injury.

[25] Pearson BC, Armitage KR, Horner CWM, Carpenter RHS (2007). Saccadometry: the possible application of latency distribution measurement for monitoring concussion. British Journal of Sports Medicine.

[26] Mullen SJ (2014). Saccadic eye movements in mild traumatic brain injury: a pilot study. The Canadian Journal of Neurological Sciences. Le Journal Canadien Des Sciences Neurologiques.

[27] Heitger MH (2009). Impaired eye movements in post-concussion syndrome indicate suboptimal brain function beyond the influence of depression, malingering or intellectual ability. Brain: A Journal of Neurology.

[28] Rayner K (1998). Eye movements in reading and information processing: 20 years of research. Psych Bulletin.

[29] Kashluba S, Hanks RA, Casey JE, Millis SR (2008). Neuropsychologic and functional outcome after complicated mild traumatic brain injury. Arch Phys Med Rehab.

[30] Arfanakis K (2002). Diffusion tensor MR imaging in diffuse axonal injury. Am J Neuroradiol.

[31] Hulkower MB, Poliak DB, Rosenbaum SB, Zimmerman ME, Lipton ML (2013). A decade of DTI in traumatic brain injury: 10 years and 100 articles later. Am J Neuroradiol.

[32] Caeyenberghs K (2011). Bimanual coordination and corpus callosum microstructure in young adults with traumatic brain injury: A diffusion tensor imaging study. J Neurot.

[33] Gooijers J, Swinnen SP (2014). Interactions between brain structure and behavior: The corpus callosum and bimanual coordination. Neurosc Biobehav Rev.

[34] Shen K (2015). Stable long-range interhemispheric coordination is supported by direct anatomical projections. Proc Natl Acad Sci USA.

[35] Makris N (2005). Segmentation of subcomponents within the superior longitudinal fascicle in humans: A quantitative, *in vivo*, DT-MRI study. Cerebral Cortex.

[36] Schulz R (2015). Parietofrontal motor pathways and their association with motor function after stroke. Brain.

[37] Allum JH, Pfaltz CR (1985). Visual and vestibular contributions to pitch sway stabilization in the ankle muscles of normals and patients with bilateral peripheral vestibular deficits. Exp Brain Res.

[38] Fitzpatrick RC, Gorman RB, Burke D, Gandevia SC (1992). Postural proprioceptive reflexes in standing human subjects: bandwidth of response and transmission characteristics. J Physiol.

[39] Simoneau GG, Leibowitz HW, Ulbrecht JS, Tyrrell RA, Cavanagh PR (1992). The effects of visual factors and head orientation on postural steadiness in women 55 to 70 years of age. J Gerontol Sep.

[40] Schumann T, Redfern MS, Furman JM, el-Jaroudi A, Chaparro LF (1995). Time-frequency analysis of postural sway. J Biomech.

[41] Wood JM (2009). Postural stability and gait among older adults with age-related maculopathy. Invest Ophthalmol Vis Sci.

[42] Posner MI, Walker JA, Friedrich FJ, Rafal RD (1984). Effects of parietal injury on covert orienting of attention. Journal of Neuroscience.

[43] Piponnier JC (2015). First- and second-order stimuli reaction time measures are highly sensitive to mild traumatic brain injuries. Journal of Neurotrauma.

[44] Lew HL, Lee EH, Pan SSL, Date ES (2004). Electrophysiologic abnormalities of auditory and visual information processing in patients with traumatic brain injury. American Journal of Physical Medicine & Rehabilitation.

[45] Madigan NK, DeLuca J, Diamond BJ, Tramontano G, Averill A (2000). Speed of information processing in traumatic brain injury: modality-specific factors. The Journal of Head Trauma Rehabilitation.

[46] Papo I, Caruselli G, Scarpelli M, Luongo A (1982). Mass lesions of the frontal lobes in acute head injuries. A comparison with temporal lesions. Acta Neurochirurgica.

[47] Baddeley, A., Eysenck M. W. & Anderson, M. C. *Memory*. 41–106 (Oxford, 2015).

[48] Ettenhofer ML, Barry DM (2016). Saccadic impairment associated with remote history of mild traumatic brain injury. Journal of Neuropsychiatry and Clinical Neurosciences.

[49] Capó-Aponte JE (2016). Visual disfucntions at different stages after blast and non-blast mild traumatic brain injury. Optometry and Vision Science.

[50] American Psychiatric Association. *Diagnostic and statistical manual of mental disorders V. (*American Psychiatric Publishing, 2013).

