# Author Correction: Long-term effects of mild traumatic brain injuries to oculomotor tracking performances and reaction times to simple environmental stimuli

**DOI:** 10.1038/s41598-018-25220-2

**Published:** 2018-05-22

**Authors:** Alessander Danna-Dos-Santos, Sambit Mohapatra, Maria Santos, Adriana M. Degani

**Affiliations:** 1 Dr. Charles T Leonard Motor Control Laboratory, University of Montana, Missoula, MT USA; 2 Neural Injury Center, University of Montana, Missoula, MT USA; 3 Department of Rehabilitation & Movement Sciences, University of Vermont, Burlington, VT USA

Correction to: *Scientific Reports* 10.1038/s41598-018-22825-5, published online 15 March 2018

This Article contains typographical errors. In the Discussion section, all instances of

“Ettenhoffer and Bardy”

should read:

“Ettenhofer and Barry”.

